# Influence of pulp industry dregs on the physical and mechanical properties of mortar

**DOI:** 10.1007/s11356-026-37821-w

**Published:** 2026-05-11

**Authors:** Luis Felipe Moreira Cesar, Agda Eunice de Souza Albas, Nelson Batista de Lima, Jorge Luis Akasaki, João Carlos Silos Moraes

**Affiliations:** 1 Department of Physics and Chemistry, São Paulo State University (UNESP), Ilha Solteira, SP Brazil; 2 Department of Physics, São Paulo State University (UNESP), Presidente Prudente, SP Brazil; 3 Center for Materials Science and Technology, Instituto de Pesquisas Energéticas e Nucleares, São Paulo, Brazil; 4 Department of Civil Engineering, São Paulo State University (UNESP), Ilha Solteira, SP Brazil

**Keywords:** Dregs, Waste reuse, Mortar, X-ray diffraction, Rietveld refinement, Thermal analysis, Pore size distribution

## Abstract

This study examined the constituents and evaluated the effects of incorporating a pulp industry residue (dregs) on the physical and mechanical properties of mortar. The properties of the mortar constituents were evaluated using thermogravimetry–differential scanning calorimetry (TG-DSC), energy-dispersive spectroscopy (EDS), X-ray Fluorescence spectroscopy (XRF), X-ray diffraction (XRD), and tests specified in standards NBR 16605 (2017), NBR 11579 (2013), and NM 18 (2012). The hardened mortar was evaluated through mechanical testing (NBR 7215 2019), water absorption testing (NBR 9778 2005), and mercury intrusion porosimetry. XRD data from dregs treated at different temperatures were analyzed using the Rietveld refinement method. The specific gravities of the cement, dregs, and sand were 3.13, 2.58 and 2.62 g/cm^3^, respectively. The main chemical elements detected in the dregs (> 1 atom%) were Ca, Mg, Mn, Si, Na, S, and Al. Thermal analysis revealed two endothermic events: the evaporation of sulfur-containing compounds and the decomposition of calcium magnesium carbonate. Lattice parameters were obtained for the Ca_0.87_Mg_0.13_(CO_3_)_2_ phase, observed in the dregs treated at 100 and 500 °C, and for the CaO and MgO phases, observed after treatment at 750 °C. To evaluate compressive strength, water absorption, and porosity, mortars were prepared under three distinct conditions: one without dregs; four with dregs added to the mixture, keeping the cement and sand contents constant; and four with dregs as a partial replacement for Portland cement, keeping the sand content constant. Compressive strength decreases with increasing dregs content, with a more pronounced reduction when Portland cement is replaced by dregs than when dregs are added. Incorporating 40 wt% dregs reduced compressive strength by 73% under the Substitution condition and 52% under the Addition condition, relative to the reference mortar without dregs. This reduction in compressive strength is associated with increased water absorption and porosity as the dregs content increases.

## Introduction

The construction industry is one of the largest consumers of raw materials worldwide and is strongly associated with environmental impacts, particularly due to the extensive use of cement-based materials. It has been reported that, in 2020, global concrete and cement production reached approximately 14 billion m^3^ and 4.2 billion tons, respectively, contributing to CO_2_ emissions on the order of 3.4 gigatons (Global Cement and Concrete Association 2024). In this context, the incorporation of industrial and agro-industrial residues into cementitious materials has gained increasing attention as a strategy to reduce landfill disposal, decrease the consumption of virgin raw materials, and contribute to the development of more sustainable construction materials within a circular-economy perspective (García et al. 2025).

Brazil is the second-largest producer of cellulose in the world, with an annual production of over 20 million tons (IBÁ 2025). Consequently, the Brazilian pulp industry generates large amounts of solid waste, mostly lime mud, dregs, grits, and biomass ash. In modern industries, lime mud is essentially recovered through advanced calcination systems, making dregs the most abundant residue resulting from cellulose production. This waste corresponds to approximately 15 kg per ton of cellulose pulp produced (Torres et al. 2020), resulting in nearly 300 thousand tons of dregs annually from Brazilian industries, most of which is disposed of in landfills. Finding sustainable solutions for this waste is a major challenge to reduce damage to the environment and, consequently, impact on the food chain and human health.

Different investigations have been carried out using paper pulp residues, such as: (a) the use of dregs as a filler in the production of geopolymeric mortars based on biomass fly ash, which is also generated during cellulose production (Novais et al. 2018, 2019); (b) the use of dregs as filler to replace commercial calcium oxide in natural rubber composites (Bittencourt et al. 2020); (c) the use of dregs-grits in mortar for wall and ceiling coating (Zanella et al. 2014); (d) the use of dregs-grits as a permeable reactive barrier for the removal of copper and sulfate in mine drainage (Farage et al. 2020); (e) the replacement of clinker with dregs-grits in the manufacture of Portland cement (Buruberri et al. 2015; Simão et al. 2019); and (f) use of dregs-grits for the correction of acidic soils (Medeiros et al. 2009; Cabral et al. 2008).

In the literature, few studies have investigated the replacement of Portland cement with dregs in mortars. Gemelli et al. (2001) examined mortars containing waste from the pulp and paper industry. They used four different materials: bottom ash, grit, dregs, and fibers. Only one sample was prepared using dregs as a replacement (2.62% relative to the dregs-free composition) for CP I-S32 cement. The incorporation of dregs into the mortar as a cement replacement reduced compressive strength by 20.4%.

Zanella et al. (2014) evaluated the durability of mortars applied to walls and ceilings containing 0, 10, and 20% dregs-grits as a substitute for river sand and lime. Their evaluation included ultraviolet radiation, salt spray, thermal degradation, and thermogravimetric tests. The way the authors reported the mortar composition suggests that dregs-grits may have been used as a substitute for lime rather than for sand. The Portland cement used was type CPIII. They concluded that incorporating 10 and 20% dregs-grits into the mortar did not affect durability under thermal degradation, thermogravimetric, or ultraviolet radiation tests, except under salt spray exposure.

Martinez-Lage et al. (2016) studied the feasibility of using fly ash and dregs in mortars and concretes by analyzing the compressive and flexural strengths after 28 days of curing. Mortars containing 10, 20, and 30% dregs were prepared to replace CEM-I 52.5 N/SR cement. They concluded that both flexural and compressive strengths decrease as the content of dregs and fly ash increases. For the mortar formulation containing 10% dregs, a 3.2% increase in compressive strength was observed compared with the reference mortar. However, the authors do not specify the number of specimens used in the mechanical tests. In contrast, decreases of 7% and 26% in compressive strength were observed for mortars containing 20% and 30% dregs, respectively.

Oliveira et al. (2023) investigated coating mortars without additives by partially replacing 10, 15, 20, and 30% of hydrated lime with dregs, evaluating properties in both the fresh and hardened states. CPII-F40 cement was used. They concluded that replacing lime with dregs, regardless of percentage, did not impair the performance of the mortars in terms of rheological, physical, or mechanical characteristics. Finally, the authors conclude that the partial replacement of hydrated lime with dregs in mortar formulations is a viable alternative that adds value to the residue, reduces costs, and mitigates environmental impacts.

Falcão et al. (2024) analyzed the effect of dregs on alkali-silica reaction in mortars. Six types of cement were used: CP II-F40, CP II-Z32, CP III-RS40, CP IV-32, CP V-ARI, and CVP-ARI RS. Dregs were added at proportions of 5, 10, and 15% relative to cement mass. Cement, sand, and water masses were kept constant. The alkali-silica reaction was evaluated by the prism method, and compressive strength was used as a control parameter for the tested mortars. The authors' main conclusions were: (a) in reference mortars, some cements produced expansions above the limit established by Brazilian standards, and in most cases, the addition of dregs increased expansions as the residue content increased; (b) cements with high additive content, such as CP IV and CP III, showed excellent ability to inhibit expansion caused by the alkali-silica reaction; and (c) cements with pozzolanic additive demonstrated lower expansion rates over time because the additive continues reacting with cement constituents.

Given the above, further research is needed to ensure the performance, durability, and safety of using cellulose waste in mortars. In this context, our group has recently begun a systematic investigation aimed at providing a sustainable application for dregs in coating and laying mortars. As the first result of this long-term effort, we present information on the characterization of the constituents (CPV-ARI cement, Tietê River sand and dregs) and on the influence of the residue on the physical and mechanical properties of the prepared mortars. Unlike previous studies, our work distinguishes itself by analyzing two distinct approaches to incorporating dregs into mortar: (i) as an addition, while keeping the cement and sand contents constant, and (ii) as a partial replacement of cement, while maintaining the sand content. This approach enabled us to evaluate the properties of the hardened mortar.

## Materials and methods

### Materials: origin and treatments

The Portland cement (CP) used in this study was CPV-ARI, a high-early-strength cement manufactured by Cimento Nacional (Arcos, MG, Brazil), which did not undergo any treatment prior to mortar preparation. This type of cement was chosen because it does not contain additives such as slag or pozzolans in its composition.

The dregs used in this study were supplied by the Suzano Papel e Celulose S.A. industrial plant in Três Lagoas (MS, Brazil). Before mortar preparation, the material was dried at 100 °C for 24 h, ground for 1 h in a jar mill using alumina balls, and then sieved to obtain particles smaller than 53 μm (ASTM 270). This pre-treatment was adopted to reduce moisture, deagglomerate the residue, and standardize its particle size before incorporation into the mortars. Similar drying and ball-milling procedures have been reported for green liquor dregs in previous studies, although with different milling times and final particle-size control procedures.

The sand used was extracted from the Tietê River by the company Porto de Areia Irmãos Brambilla (Pereira Barreto, SP, Brazil). Before use, the sand was dried at 100 °C for 24 h and then sieved according to NBR 7214 (2015). Consequently, the sand used in this study consists of grains ranging from 0.15 to 2.4 mm.

### Raw materials characterization

The specific mass of the precursors (cement, sand and dregs) was determined according to the procedure recommended by NBR 16605 (2017), which uses a Le Chatelier volumetric flask. Deionized water was used in the tests with dregs and sand, while kerosene was used for the cement tests. Three tests were performed for each precursor. The fineness modulus and loss on ignition of the cement were evaluated following the methodology prescribed in NBR 11579 (2013) and NM 18 (2012), respectively.

The structural properties of the dregs were investigated using TGA/DSC, energy dispersive X-ray spectroscopy (EDX), X-ray fluorescence spectroscopy (XRF), and X-ray diffraction (XRD) techniques. Thermal behavior was obtained with a simultaneous thermal analyzer from TA Instruments, model Q600 SDT, operating from room temperature to 1100 °C at a heating rate of 10 °C/min under nitrogen atmosphere. Chemical elements identification in the dregs was performed using an EDX detector coupled to a Zeiss scanning electron microscope (SEM), model EVO-LS15. Diffractograms were obtained with a Shimadzu diffractometer, model XRD-6000, using Cu-Ka radiation, at room temperature, with a Bragg angle between 20 and 70°. The particle size distribution of cement was determined using a HORIBA LA-960 laser analyzer, whereas that of sand was determined in accordance with NBR 7211 (2022) and NBR 7214 (2015).

### Mortar preparation

Nine different mortar compositions were prepared (Table 1): one without dregs (Ref), four with dregs added to the mixture (A), and four with dregs used as a partial replacement for CP (S), in which the sand content was kept constant at concentrations of 5, 10, 20, and 40 wt%. The sand mass was kept constant, and the water-to-fines ratio (cement + dregs) was adjusted for each composition to achieve a flow of (260 ± 5) mm, as recommended by NBR 16541 (2016).

**Table 1:** Mixture composition of the mortars

Sample name	Cement (g)	Dregs (g)	Sand (g)	Water/fines ratio
Ref	900	0	2700	0.55
A05	900	45	2700	0.50
A10	900	90	2700	0.50
A20	900	180	2700	0.48
A40	900	360	2700	0.50
S05	855	45	2700	0.58
S10	810	90	2700	0.55
S20	720	180	2700	0.55
S40	540	360	2700	0.58

The incorporation of dregs into the mixture slightly altered the water/fines ratio, as shown in the table. In the Addition condition, the required water/cement ratio required was lower (from 0.48 to 0.5) than that of the sample without dregs (0.55). In contrast, under the partial replacement condition, where CP was substituted by dregs, it was necessary to increase the ratio to 0.58 in two of the four proportions tested (5 and 40%).

Five test specimens were prepared for the compressive strength tests and three for the water absorption tests, according to NBR 7215 (2019) and NBR 9778 (2005), respectively. Cylindrical metal molds measuring 5 cm in diameter and 10 cm in height were used. Immediately after molding, the specimens were stored in a chamber at 100% relative humidity and a temperature of (23 ± 2) ºC. After 24 h, they were demolded and kept in the chamber until completing 28 days of curing.

### Physical and mechanical properties of hardened mortars

Mechanical tests were performed using an EMIC universal testing machine, model DL30000M, equipped with a 300 kN load cell and operating at a rate of (0.25 ± 0.05) MPa/s. Water absorption was determined in accordance with NBR 9778 (2005), using an oven (Odontobras – SP, Brazil), model EL1.1, and a digital balance with a precision of 0.1 g. First, the specimens were weighed after drying at (105 ± 5) ºC for 72 h. They were then immersed in water for the same period and weighed again. Water absorption was calculated using the expression below, where *m*_*i*_ and *m*_*s*_ correspond to the dry mass and water-saturated mass of the specimen, respectively.

$${Abs}_{H2O}=\left(\frac{{m}_{s}-{m}_{i}}{{m}_{i}}\right)\times 100$$

Porosity was evaluated using a mercury intrusion porosimeter (Autopore III—Micromeritics) within a pressure range of 0 to 414 MPa. The minimum detectable pore size was 3 nm, corresponding to the maximum applied pressure. A 1 cm^3^ sample of each mortar composition was extracted from the specimens used in the water absorption test. Prior to analysis, all samples were degassed under vacuum at a pressure below 50 Pa.

### Statistical analysis

Statistical analysis was performed using SigmaPlot software (Systat Inc, San Jose, CA, USA), version 12.0. The significance level was set at *p* < 0.05. Water absorption and compressive strength data showed a normal (Shapiro–Wilk) and homogeneous (Levene's test) distribution and were subjected to one-way analysis of variance, followed by Fisher LSD test.

## Results and discussion

### Raw material characterization

The specific masses (mean ± SD) obtained for the cement, dregs, and sand were (3.13 ± 0.02), (2.58 ± 0.01), and (2.62 ± 0.01) g/cm^3^, respectively. The CPV-ARI cement exhibited a fineness index of 5.9 and a loss on ignition of 5.7%, both in accordance with the limits established by standards NBR 11579 (2013) and NM 18 (2012), respectively.

**Fig. 1:**
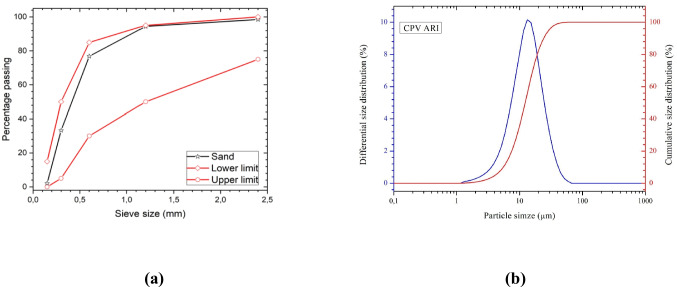
Particle size distribution curve of (**a**) sand and (**b**) Portland cement

Before the sieving step used to prepare the mortar mixtures, the particle size distribution of the natural river sand was determined in accordance with NBR 7211 (2022) and NBR 7214 (2015), using a set of five sieves with mesh sizes of 2.4, 1.2, 0.6, 0.3, and 0.15 mm. The cumulative passing curve obtained (Fig. 1a) showed values of 98.5% at 2.4 mm, 94.4% at 1.2 mm, 76.8% at 0.6 mm, 33.3% at 0.3 mm, and 2.1% at 0.15 mm.

These results indicate that approximately 75% of the sand particles fall within the size range of 0.15 to 0.6 mm. Based on the cumulative retained percentages, the natural river sand presented a fineness modulus of approximately 1.95. According to NBR 7211 (2022), this value falls within the lower usable grading zone for fine aggregates. Furthermore, the particle size distribution of the Tietê River sand lies within the lower and upper limits defined by the standard. In particular, the low passing percentage at 0.15 mm (2.1%) indicates a low fines content, which is generally desirable for laying mortars, as it helps reduce excessive water retention and minimizes the risk of shrinkage associated with an excess of fines.

Figure 1b shows the particle size distribution of Portland cement obtained using the laser diffraction technique. This technique was selected because it enables the evaluation of particle size distribution over a wide micrometer range, providing more accurate characterization of fine materials such as Portland cement. The analysis indicated a median diameter (D50) of approximately 12.2 µm, with D10 and D90 values of 5.5 µm and 23.7 µm, respectively.

**Fig. 2:**
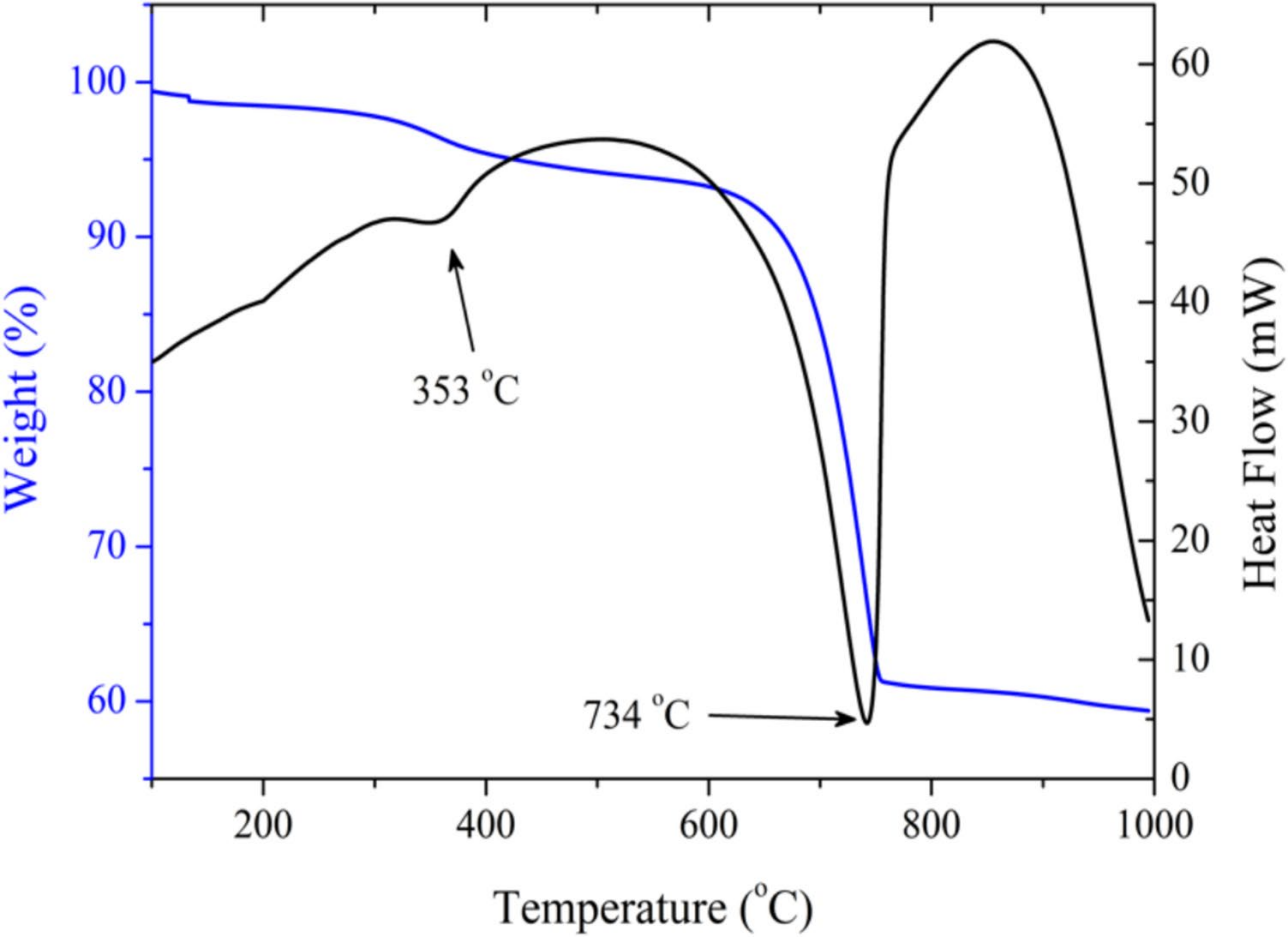
DSC (heat flow) and TGA (mass-loss) curves of dry dregs powder

Figure 2 shows the TGA/DSC thermograms obtained for the dry dregs. In the TGA curve (blue), three mass-loss events can be observed: from 50 to 200 °C, from 200 to 550 °C, and from 550 to 1000 °C. The first event corresponds to a slight mass loss (1%), attributed to the evaporation of residual water. The second event, corresponding to a 4.5% mass loss, may be due to the decomposition of sulfur-containing compounds resulting from the chemical recovery stage of the kraft process, and is associated with the endothermic peak at 353 °C in the DSC curve (black). This endothermic event was also reported by Andesanya et al. (2025), who attributed it to the dehydroxylation of brucite; however, as shown below, this phase is not observed in our XRD data. Martins et al. (2007) and Torres et al. (2020) associated this endothermic peak with gypsum dehydration; however, complete gypsum dehydration generally occurs below 200 °C.

Elemental analysis by EDX shows the presence of small amounts of sulfur and sodium in the dregs (Table 2), indicating that, despite the high efficiency of the kraft chemical recovery process, it is not fully complete. Among the possible sulfur compounds formed in this process is sodium thiosulfate. Despite its greater solubility compared to sulfides, sulfates, and sulfites, sodium thiosulfate can precipitate in highly alkaline media, and its decomposition occurs between 300 and 400 °C (Padamurthy et al. 2019; McAmish and Johnston 1976).

**Table 2:** Main chemical elements detected in the dregs by EDX spectroscopy

Chemical element	Treated to 100 °C (atomic%)	Treated to 750 °C (atomic%)
O	51.3	38.8
C	8.4	3.3
Ca	20.6	33.8
Mg	10.3	12.6
Mn	2.7	3.1
Si	2.2	2.7
Na	1.3	1.7
S	1.6	2.1
Al	0.9	1.1
Fe	0.7	0.8

**Fig. 3:**
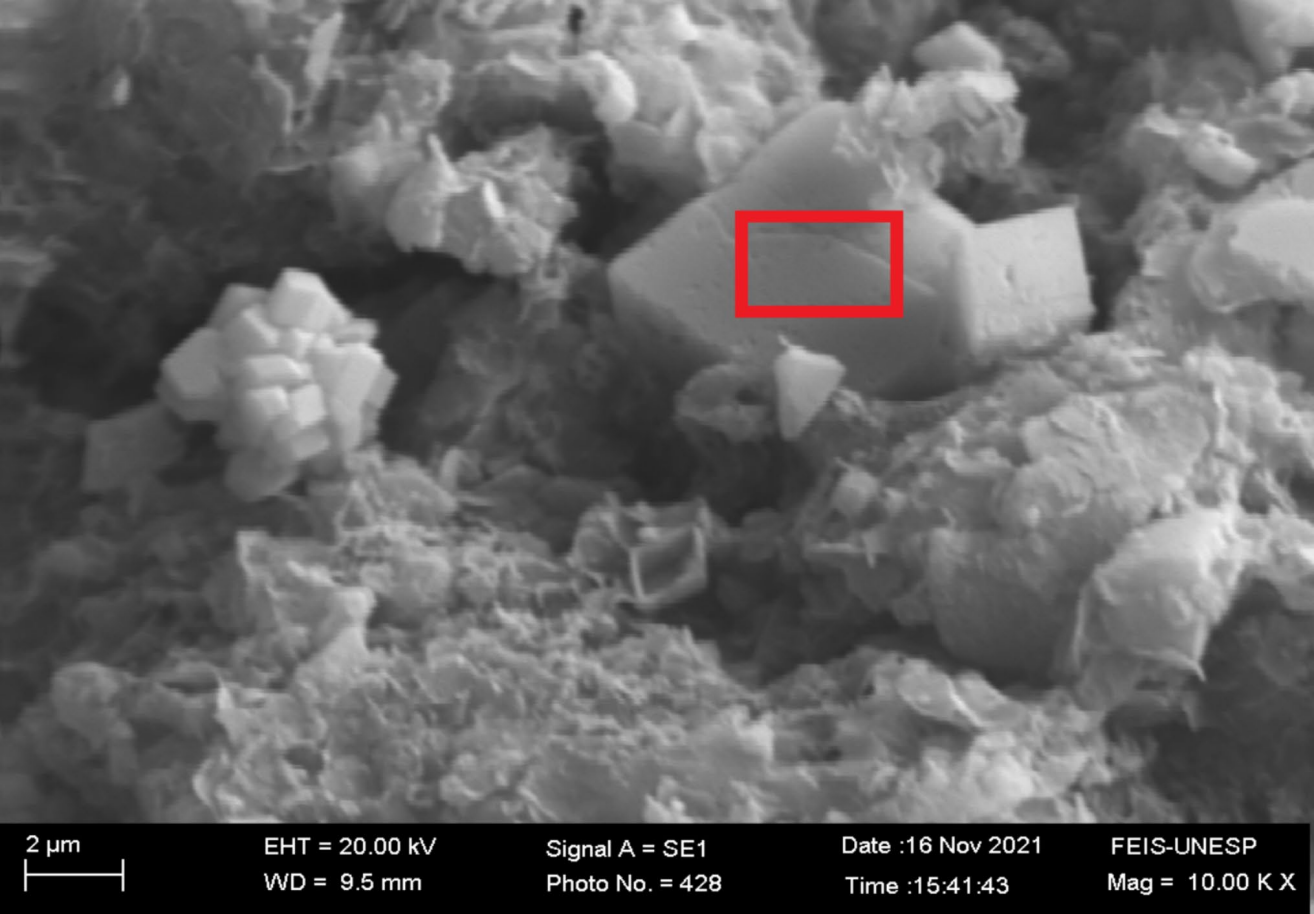
SEM image of dry dregs powder at 10,000x magnification, highlighting an area on a crystalline structure

The last mass-loss event (34.4%) results from the decomposition of calcium carbonate, which releases CO_2_ and forms calcium oxide (Santos et al. 2019). This decomposition corresponds to the endothermic peak observed at 734 °C in the DSC curve. A decrease in the mass percentage of C and O can also be observed in the EDX analysis (Table 2), as expected given that calcium carbonate is known to be the main constituent of the dregs. The SEM image of the dry dregs in Fig. 3 shows crystalline structures of various sizes. The analysis performed in the highlighted area reveals predominantly the presence of C, O, and Ca, confirming that the Ca in these crystalline structures is present in the form of carbonate.

**Fig. 4:**
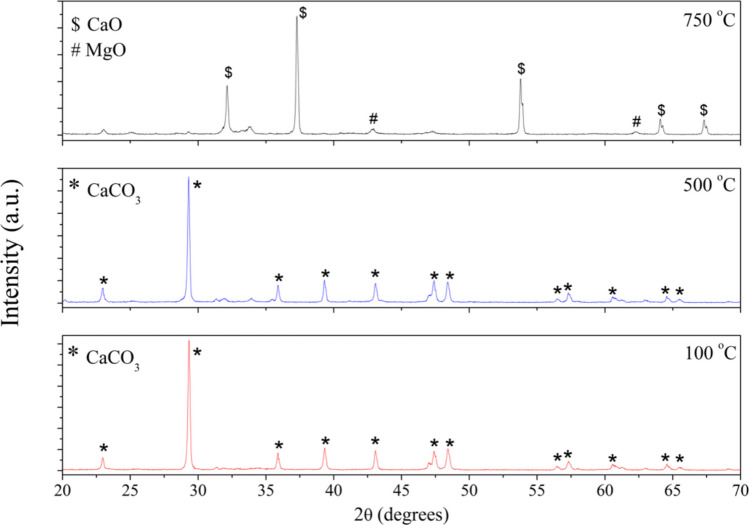
XRD patterns of dregs subjected to three different heat-treatment conditions: 100 °C for 24 h, 500 °C for 1 h, and 750 °C for 2 h

To observe the phase transformations, the dried dregs powder (treated at 100 °C for 24 h) was subjected to two additional heat treatments: 500 °C for one hour (a temperature between the two endothermic transitions observed in Fig. 2) and 750 °C for two hours (above the endothermic transition centered at 734 °C). Figure 4 shows the diffractograms of dregs powders treated at three different temperatures. In the first two diffractograms, corresponding to treatments at 100 and 500 °C, only the calcium carbonate phase (ICSD #73446) was observed. In contrast, the powder treated at 750 °C, exhibited the CaO (ICSD #51409) and MgO (ICSD #61325) phases.

**Fig. 5:**
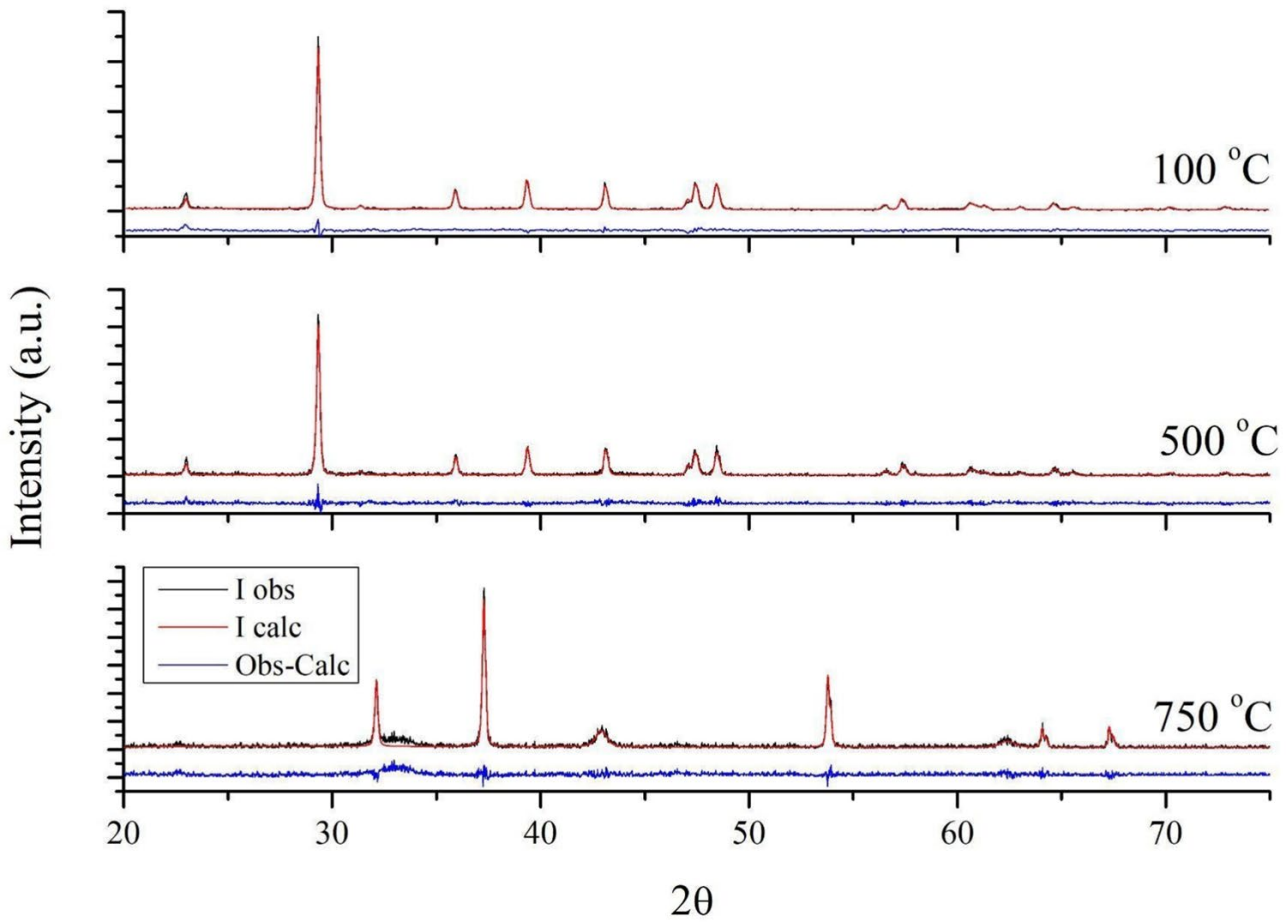
Rietveld refinement plot of the dregs treated at different temperatures, showing the observed (black) and calculated (red) diffractograms, as well as their difference (blue)

The absence of the crystalline magnesium phase in the dregs powders treated at 100 and 500 °C suggests that Mg may be partially substituting Ca in the calcium carbonate structure. Therefore, the diffractograms were analyzed by the Rietveld refinement method using the GSAS-II software package. For the diffractograms corresponding to 100 and 500 °C, a standard crystalline phase of calcium-magnesium carbonate was used (ICSD card #40108). During refinement, the occupancy parameters of the Ca and Mg atoms were set at 0.87 and 0.13, respectively. These values were estimated from XRF data, using a calibration curve constructed with standard mixtures of CaO (Sigma-Aldrich, 99 + %) and MgO (Sigma-Aldrich, ≥ 99%) in different proportions. Figure 5 shows the Rietveld output for the analyzed dregs powders, and the structural parameters extracted from the refinement are summarized in Table 3.

**Table 3:** Lattice parameters obtained from the Rietveld refinement of the dregs powder heat-treated at three different temperatures

T (^o^C)	Lattice parameters (Ǻ)			Weight fraction		
	Ca_0.87_Mg_0.13_(CO_3_)_2_	CaO	MgO	Ca_0.87_Mg_0.13_(CO_3_)_2_	CaO	MgO
100	a = b = 4.98904 c = 17.0616 α = β = 90 γ = 120			1	0	0
500	a = b = 4.98839 c = 17.07828 α = β = 90 γ = 120			1	0	0
750		a = b = c = 4.81512 α = β = γ = 90	a = b = c = 4.21342 α = β = γ = 90	0	0.864	0.136

### Mortar in the hardened state

**Fig. 6:**
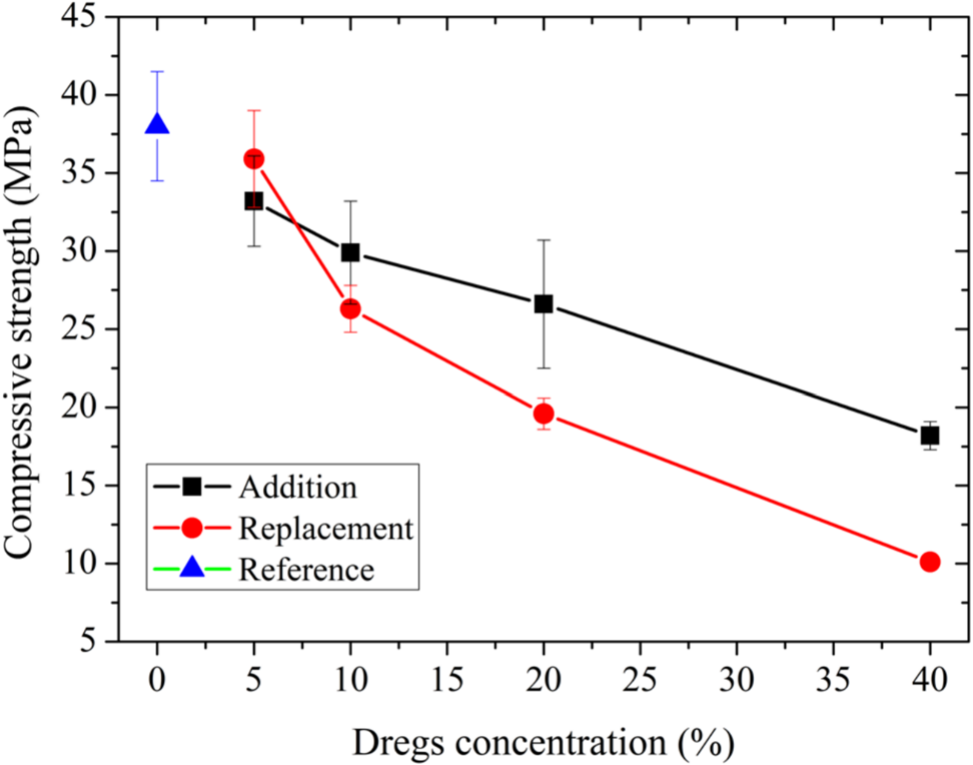
Compressive strength of hardened mortars without dregs (blue triangle), with dregs added to the mixture (black squares), and with dregs partially replacing Portland cement (red circles)

The influence of dregs on the composition of mortars was evaluated based on their physical and mechanical properties in the hardened state. Figure 6 shows the results of the compressive strength tests for the mortars without dregs (Reference) and with dregs incorporated by addition and replacement conditions. Overall, compressive strength decreased as the dregs content increased, regardless of the incorporation method. This trend is more pronounced in the replacement condition, suggesting that dregs do not participate in the hydration process. In this condition, the reduction in cement limits the formation of binding phases such as calcium silicate hydrate (C–S–H), which is essential for strength development. In the addition condition, although the cement content has been maintained, the dregs may act as an inert material, diluting the matrix without contributing reactivity. Statistically, no significant differences were observed between Ref and S05, A5 and S5, A5 and A10, A10 and A20, A20 and S10, and A40 and S20.

In contrast, Falcão et al. (2024) observed no significant difference in compressive strength when 5—15 wt% dregs were added to mortar prepared with CPV-ARI cement. This difference in behavior may be attributed to the richer composition used by the authors (cement/sand = 1:2.25), the fixed water/cement ratio at 0.47, and the difference in dreg granulometry.

Oliveira et al. (2023) also did not observe significant differences in compressive strength or water absorption in mortar formulations. In contrast to our study, their formulations involved replacing hydrated lime with dregs at levels of 10, 15, 20, and 30%. Their findings are consistent, since the cement and sand contents were kept constant and the primary role of hydrated lime is to improve the workability, adhesion, and plasticity of the mortar.

Martinez-Lage et al. (2016) also observed a decrease in compressive strength when cement was replaced with dregs in mortar formulations. However, the reduction observed in our study was much more pronounced. This greater decrease can be attributed to the higher water-to-(cement + dregs) ratio used in our mixtures compared with that reported by Martinez-Lage et al. As this ratio increases, the compressive strength tends to decrease, since a higher water content generally leads to increased porosity in the hardened mortar.

**Fig. 7:**
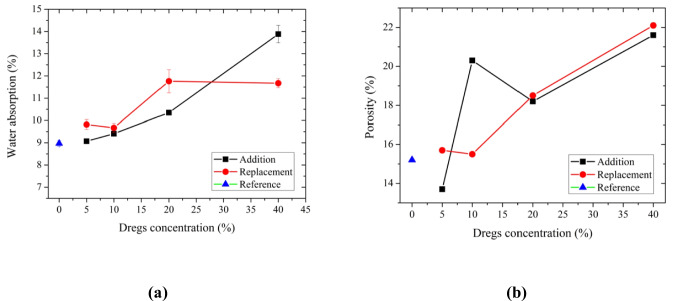
(**a**) Water absorption and (**b**) porosity of hardened mortars without dregs (blue triangle), with dregs added to the mixture (black squares), and with dregs partially replacing Portland cement (red circles)

Opposite to the behavior observed for compressive strength, the water absorption results (Fig. 7a) show an increase in absorption as the dregs content increases, with a more pronounced effect in the addition condition. Statistically, only A05 did not differ significantly from the control composition (Ref). Similar behavior was observed in porosity measurements (Fig. 7b), with pore volume increasing as the dregs content increased.

**Fig. 8:**
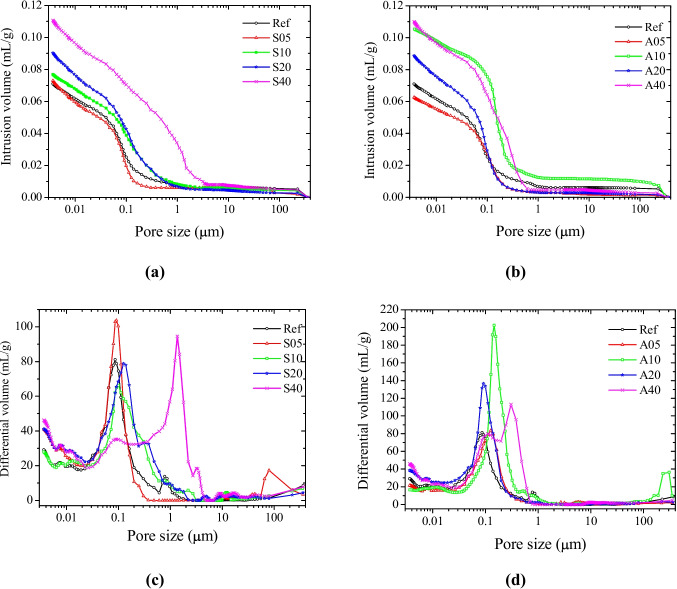
Evolution in pore-size distribution in hardened mortars prepared under the replacement (**a**, **c**) and addition (**b**, **d**) conditions

The evolution of pore distribution as a function of dregs content after 28 days of curing is shown in Figs. 8a and c (replacement condition) and 8b and 8 d (addition condition). In general, regardless of the method of incorporation, the pore size distribution in Ref showed a narrow peak between 0.02 and 0.61 µm, centered at 0.09 µm (Figs. 8c and d), corresponding to C–S–H gel pores. Increasing the dregs content shifted this peak toward larger pores, likely due to the weakening of the interfacial transition zone (ITZ) between the cementitious matrix and the sand grains (Chen et al. 2024; Scrivener et al. 2004). Structurally, the ITZ is less dense and more porous compared with the surrounding cement matrix. The peak shift indicates that the powder does not contain a sufficiently fine particle size for the dregs to act as a filler, as their particle size was not controlled for this purpose.

Unlike the other compositions, two distinct pore distributions were observed in A40 and S40: one corresponding to smaller pores within the cement matrix, and another associated with larger pores from the ITZ and dregs particles. This confirms that dregs act as inert particles, generating larger voids after the evaporation of retained water. Furthermore, the relative proportion of these two pore populations is greater in A40 than in S40, indicating that in the replacement condition the population of smaller pores is reduced due to the lower cement content, corroborating the interpretation that these smaller pores are associated with the cementitious matrix.

Finally, in addition to these factors, the lower specific mass of dregs (2.58 g/cm^3^) compared with cement (3.13 g/cm^3^) contributed to changes in packing density and pore structure, resulting in the observed compressive strength behavior as the residue content increased.

The results presented in this study encourage the continuation of this research with the aim of developing mortars containing dregs that are suitable for application on walls and ceilings. Future work will focus on mortar compositions incorporating dregs under the addition condition, as well as on the evaluation of their adhesion and fire resistance properties. In addition to the environmental advantages associated with waste reuse, the incorporation of dregs into mortars may provide economic benefits by reducing landfill disposal requirements and, depending on the incorporation strategy, partially decreasing the consumption of conventional raw materials. Therefore, the use of this residue may contribute to its valorization within a circular economy framework.

## Conclusion

In this study, the influence of dregs on the physical and mechanical properties of mortars was evaluated. Dregs were incorporated into the mortar composition in two different ways: by direct addition to the mixture and by partial replacement of cement. Two endothermic events were observed in the thermogravimetric curve: the first was probably associated with sodium thiosulfate, and the second with the decomposition of calcium and magnesium carbonate, releasing CO_2_ and forming CaO and MgO. No significant change was observed in the lattice parameters of the Ca_0.87_Mg_0.13_(CO_3_)_2_ phase in the dregs powders treated at 100 and 500 °C. In the diffractogram of the powder treated at 750 °C for 2 h, only the CaO and MgO phases were observed, with weight fractions of 86.4% and 13.6%, respectively.

Increasing the dregs content in the mortar composition, regardless of the incorporation method (addition or replacement), increases the pore volume and alters the pore-size distribution, resulting in a decrease in compressive strength. Despite the significant reduction in strength for dregs contents of 20 and 40%, this does not preclude the use of the residue in mortar for certain civil construction applications, such as laying and coating.

The main contribution of this study is to provide a better understanding of the effect of pulp industry dregs on mortar behavior, particularly with regard to porosity development, water absorption, and compressive strength. These findings contribute to the assessment of this residue as a potential alternative raw material for more sustainable cement-based materials. However, further studies are needed to demonstrate its full potential for use in mortars for wall and ceiling applications, where compressive strength is less critical, but good workability, adhesion, and adequate permeability are required.

## Data Availability

The authors declare that the data supporting the findings of this study are available within the paper and its Supplementary Information files. Should any raw data files be needed in another format they are available from the corresponding author upon reasonable request. Source data are provided with this paper.
